# Performance of Anticorrosive Paint Systems for Carbon Steel in the Antarctic Marine Environment

**DOI:** 10.3390/ma16165713

**Published:** 2023-08-21

**Authors:** Rosa Vera, Margarita Bagnara, Rodrigo Henríquez, Lisa Muñoz, Paula Rojas, Andrés Díaz-Gómez

**Affiliations:** 1 Instituto de Química, Facultad de Ciencias, Pontificia Universidad Católica de Valparaíso, Av. Universidad 330, Placilla (Curauma), Valparaíso 2373223, Chile; margarita.bagnara@pucv.cl (M.B.); rodrigo.henriquez@pucv.cl (R.H.); lisa.munoz@pucv.cl (L.M.); 2 Facultad de Ingeniería y Ciencias, Universidad Adolfo Ibáñez, Diagonal Las Torres 2640, Santiago 7941169, Chile; paula.rojas.s@uai.cl

**Keywords:** Antarctic, atmospheric corrosion, organic coatings, carbon steel

## Abstract

This study evaluated the behavior of three paint systems exposed to the Antarctic marine environment for 45 months compared to a control of uncoated carbon steel with a determined corrosion rate. At the study site, all environmental conditions, solar radiation, and the concentration of environmental pollutants (${\mathrm{Cl}}^{-}$ and SO_2_) were evaluated. The paint systems differed in terms of the primer and top coat. Coated samples were studied before and after exposure. They were evaluated visually and using SEM to determine adhesion, abrasion, and contact angle; using the Evans X-Cut Tape Test; using ATR-FTIR spectroscopy to analyze the state of aging of the top layer; and using electrochemical impedance spectroscopy (EIS) for coat protection characterization. The corrosion rate obtained for steel was 85.64 µm year^−1^, which aligned with a C5 environmental corrosivity category. In general, the evaluation in the period studied showed the paint systems had good adhesion and resistance to delamination, without the presence of surface rust, and exhibited some loss of brightness, an increase in the abrasion index, and a decrease in the percentage of reflectance due to aging. EIS showed good protection capability of the three coating schemes. In general, this type of paint system has not previously been evaluated in an extreme environment after 45 months of exposure to the environment. The results showed that the best behavior was found for the system whose top layer was acrylic–aliphatic polyurethane.

## 1. Introduction

Although the number of investigations in the field of corrosion in cold climates continues to increase, it is less than that in areas with other types of environments [1]. Mikhailov et al. conducted corrosion tests on various metals and alloys in cold and very cold climate regions, such as the Antarctic, subarctic areas, and the Russian Far East. Their findings indicated that metal corrosion occurs even in extremely cold climates with sub-zero temperatures. Notably, carbon steel exhibited the highest susceptibility to corrosion under low-temperature conditions, particularly in proximity to the sea, due to chloride contamination [1]. Similarly, investigations conducted in Antarctic coastal sites, as part of the Iberoamerican Map of Atmospheric Corrosivity (MICAT) [2], reported corrosion rates higher than expected for mild steel, zinc, and copper, yet lower than those observed in temperate atmospheres. It is important to consider that in these areas, the metal is primarily covered with a layer of ice during most of the exposure period and subjected to intense winds and limited precipitation [3]. Subsequent to MICAT, studies were conducted in various Iberoamerica countries to evaluate different paint systems (PATINA) for steel corrosion protection [4].

The selection of corrosion protection methodologies, such as paint systems, is crucial. Momber et al. investigated the corrosion of steel at a temperature of −60 °C in an environmental chamber for a duration of 25 days. The steel was protected by a paint system consisting of two layers of epoxy and a top layer of aliphatic polyurethane, with a total thickness of 1400 µm. At low temperatures, organic systems exhibit changes in their response, transitioning between plastic–elastic and elastic–plastic behavior, while also experiencing increased rigidity modulus, altered hardness, and susceptibility to cracking. Under these conditions, the adhesion of the paint system to the substrate tends to decrease, along with its impact resistance, abrasion resistance, and corrosion resistance [5,6,7].

Additionally, Sun et al. conducted studies on epoxy coatings containing Fe_3_O_4_ nanoparticles, aiming to establish a correlation between corrosion protection at room temperature and below room temperature and the mechanical properties of the coatings. The results revealed that the addition of nanofillers enhances the adhesion, tensile strength, and low-temperature flexibility of the epoxy coating. Furthermore, optimal dispersion of the fillers contributes to improved coating performance [8]. Conversely, three organic coatings were subjected to aging in a simulated offshore environment in the Arctic. The results demonstrated a shorter lifespan for all three coatings compared to those observed in low-temperature or UV aging tests. This performance discrepancy is attributed to the synergistic effect of UV radiation, salt spray, and temperature variations (ranging from 60 °C to −40 °C) within the environment. Notably, the polyurethane coating exhibited superior anti-corrosion performance compared to the epoxy coating system. The exposure process in this medium involves UV radiation-induced embrittlement, salt spray accelerating water absorption, and temperature variations causing volume changes in the corrosive medium within the coatings [9]. Further studies indicate that the application of two-component organic systems (acrylic resins cured with aliphatic polyurethane) on carbon steel and galvanized steel, with thicknesses ranging from 60 to 100 µm, results in blistering and reduced coating hardness over time [10]. Generally, the behavior of a paint system can be influenced by temperature variations, which affect scratch adhesion. At temperatures below room temperature, the paint becomes less ductile [11].

Next, studies of certain polyurethane (PU) membranes have evaluated degradation behavior over time under artificial environmental conditions. These investigations revealed that the decomposition of PU membranes is a complex process involving physical and chemical phenomena, such as chain scission, chain rearrangement, and cross-linking [12]. For instance, polyurethane-coated steel probes with varying thicknesses (60, 80, and 100 µm) were subjected to a marine environment, and their degradation was evaluated using electrochemical impedance. The deterioration of the coatings over time was correlated with changes in mechanical properties, including microhardness and adhesion. Notably, the polyurethane coating exhibited strong adhesion to the steel substrate [13].

On another note, the addition of nanoparticles (SiO_2_, Zn, Fe_2_O_3_, and clay) to epoxy coatings applied to steel and exposed to a NaCl solution has shown improved corrosion behavior and increased Young’s modulus of the epoxy coating. This enhancement is attributed to the improved microstructure of the coating matrix [14]. However, epoxy coatings, in general, exhibit inferior anticorrosion performance at low temperatures due to embrittlement. The freezing process can accelerate the diffusion of aggressive ions through the polymeric matrix [15]. Zhang et al. conducted a 20-year study on the behavior of epoxy coatings at sites in Wanning. Although the coating appeared intact, it had lost its protective properties, as confirmed by electrochemical impedance measurements. The deterioration was attributed to the presence of cracks in the coating [16].

Other studies, which made use of accelerated salt spray chamber tests conducted on three-layer coatings, found that a Zn-rich epoxy coating as the first layer (providing cathodic protection), an epoxy intermediate layer, and a polyurethane top layer had good performance against steel corrosion. The polyurethane top layer, characterized by a cross-linked structure with compact packing, reduces free volume and impedes the ingress of water molecules [17]. Additionally, the incorporation of metallic compounds into Zn-rich paints significantly improves their corrosion protection capabilities for the underlying metal [18].

Presently, nanotechnology has opened avenues for the development of superhydrophobic nanocomposite coatings with excellent properties for protecting metallic materials against corrosion. Epoxy coatings, due to their widespread industrial usage, have garnered significant research interest in this area, and the utilization of superhydrophobic coatings appears to be a promising solution for protecting steel from the corrosion process [19,20].

In the present study, three paint systems typically used in Chile for carbon steel structures exposed to marine environments were evaluated for the first time in the Chilean Antarctic territory for 45 months (average temperature below 0 °C; see Table S1).

The paint schemes are made up of the following:

- A primer layer, which is in contact with the metal and fulfills the function of generating the necessary adhesion or anchorage to support the rest of the paint layers without any sagging of the complete system. In addition, the primer layer has additives that act as passive inhibitors, which protect the metal once it is exposed to the corrosive environment.
- An intermediate layer, which provides volume or thickness to generate a broader physical barrier effect and hinder the advance of humidity and/or meteorochemical agents toward the metal surface.
- A top coat, which fulfills a special and important function, namely, to protect the entire paint system from the elements. This top layer must have a high resistance to humidity, chemical agents, UV radiation, and abrasion, among other qualities. It is the first protection shield that allows the underlying layers to fulfill their function.

These schemes differed in terms of primer coat (Zn-rich epoxy and primer) and finish (epoxy and polyurethane).

## 2. Experimental Procedures

### 2.1. Materials

#### 2.1.1. Metal Substrate

To apply the paint systems, A-36 carbon steel probes of dimensions of 10 × 10 × 0.3 cm were used as the base metal. The chemical composition of the steel was determined using optical emission spectrometry with a SPECTROMAXx instrument (LMX10, Kleve Germany). The chemical composition of A-36 steel is presented in Table 1.

#### 2.1.2. Paint Systems

The steel samples were subjected to the white metal grade shot blasting process (SSPC-SP10). Paint systems were applied to steel probes using Airases Graco 56:1 equipment. During the application process, environmental conditions—such as ambient humidity, ambient temperature, substrate temperature, and dew point—were carefully controlled. Additionally, parameters such as wet thickness, dry thickness, and porosity of the painted samples were monitored.

**Figure 1:**
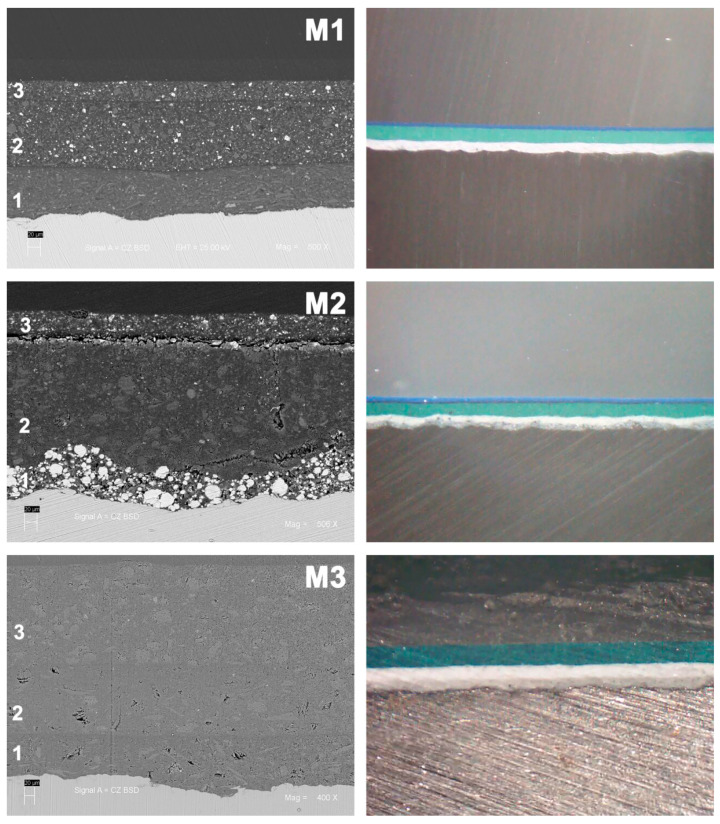
Initial slice appearance of painted steel with the different paint schemes (SEM and magnifying glass): 1: primer, 2: intermediate, 3: top coat.

Three paint systems, commonly used in marine environments, were evaluated according to the international standard ISO 12944-5 [21], which specifies systems with high durability. These systems were designated as M1, M2, and M3, and their characteristics are detailed in Table 2. To visually analyze the distribution of the different paint layers for each system, slice photographs were obtained (Figure 1) using a stereoscopic magnifying glass and scanning electron microscopy (SEM) on a HITACHI SU3500 (Tokyo, Japan) coupled with a Bruker XFlash 410 M device (Berlin, Germany). The porosity of the paint schemes was determined using the holiday detector and by applying the ASTM G62 standard [22]. However, no porosity was identified that could generate micro-structural defects in the schemes before being exposed.

The three paint systems investigated differed primarily in terms of the primer and top coat layers, while the intermediate coat remained consistent across all systems. Specifically, the M1 and M3 systems employed the same self-priming epoxy primer, whereas the M2 system incorporated a zinc-rich epoxy primer. In terms of the top coat, M1 utilized an aliphatic acrylic polyurethane, M2 utilized an aliphatic polyurethane, and M3 employed an epoxy top coat. The intermediate coat for all three systems was an epoxy enamel. While all three layers of the paint system provide protection against environmental factors, it is the top coat that directly faces the external environment.

Table 3 presents some characteristics of the paints used in the top coat layer. In Figure 1, the M1 and M2 samples exhibit top coat thicknesses of 31.11 µm and 38.95 µm, respectively, and the thickness of sample M3 was 170.33 µm.

### 2.2. Field-Test Procedures

**Figure 2:**
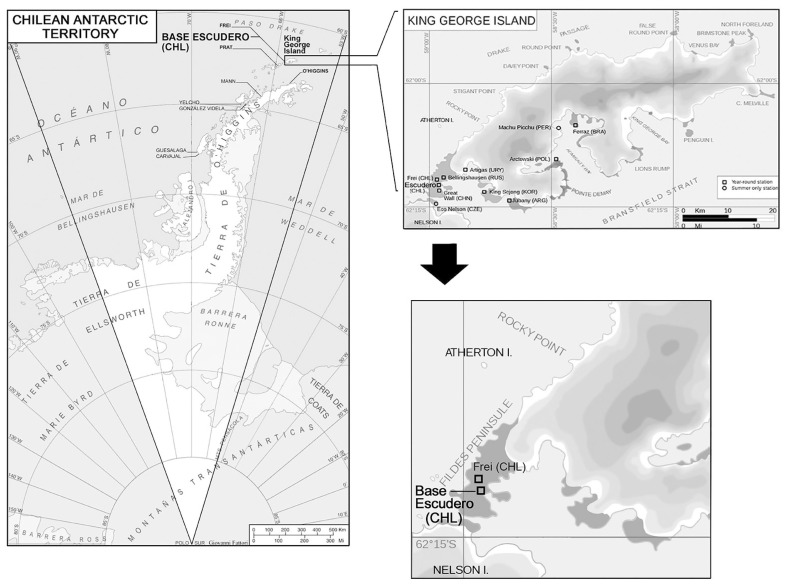
Location of test station.

The study was conducted at the Professor Jorge Escudero base, situated on the Fildes Peninsula, latitude 62°12′57″ S and longitude 58°57′35″ W, on King George Island, part of the South Shetland Islands in the Chilean Antarctic Territory. The test site was located approximately 100 m away from the sea (Figure 2). The study spanned 45 months, starting in March 2014 and concluding in December 2017.

For the experiment, both unpainted steel specimens and painted specimens were installed on galvanized steel racks. These specimens were positioned at a 45° angle to the rack and were separated by plastic insulators, adhering to the standards specified in ISO 9223 and ASTM G-50 (Figure S1) [23,24].

### 2.3. Meteorological and Pollution Data

The meteorological station installed at Escudero Base provided monthly data on temperature (T), relative humidity (HR), amount of rainfall, wind speed, and solar radiation. Devices were also installed to take bimonthly measurements of chloride and sulfur dioxide content in the air (Figure S1). The wet candle method was used to measure atmospheric chlorides and results are expressed in mg Cl^−^ m^−2^ day^−1^ [25]. For measurements of SO_2_, the lead dioxide candle method was used and the result is expressed in mg SO_2_ m^−2^ day^−1^ [25].

### 2.4. Corrosion Testing of Bare Samples

The corrosion rate of the material was evaluated every 6 months by measuring mass loss in triplicate (ASTM G50) [23]. The morphology of the attack was observed under a scanning electron microscope (SEM) using a Hitachi SU 3500 with a 410-M EDAX analyzer for elemental characterization.

### 2.5. Evaluation of Paint Systems

The painted samples, both with and without exposure, underwent several evaluations to assess their properties.

First, the thickness of the paint coatings was measured using an Elcometer 456 digital tester (Houston, TX, USA) following the ISO 2808 standard [26]. Additionally, the tensile bond strength was determined using an Elcometer Model 106 tester (Houston, TX, USA), according to the ASTM D4541-17 specification [27].

Visual evaluations were conducted to assess blistering, following ASTM-D714 [28], and using Evans X-Cut Test. The brightness of the paint coatings was measured using a BYK Gardner micro-TRI-gloss Glossmeter (Essen, Germany), while color measurements were obtained using a Data color 650 TM Spectrum device. FTIR-ATR spectra analysis was performed on a Perkin Elmer Spectrum Two instrument (Tokyo, Japan).

The contact angle (CA) was measured to evaluate surface wettability. A Kruss-Scientific model DSA25S goniometer (Hamburg, Germany) was controlled by ADVANCE software (KRÜSS) and the procedure was undertaken by depositing 8 μL drops of water or diiodomethane on the surface under study.

To assess the abrasion resistance of the paint coatings, mechanical tests were conducted according to ASTM D-4060 [29] using a TABER model 5131 Abraser equipped with CS-17 abrasion wheels and applying a load of 1000 g. These tests were performed on three evaluated schemes and repeated in triplicate. The resulting abrasion index represents the amount of coating lost per 1000 abrasion cycles, expressed in milligrams (mg).

Finally, resistance of paint systems was measured using electrochemical impedance spectroscopy (EIS). Measurements were performed every 6 months using an AUTOLAB model 302A. The measurements were conducted at the corrosion potential, with a frequency of 0.1 Hz, in a Na_2_SO_4_ 0.1 M solution at a temperature of 20 ± 2 °C. A three-electrode cell configuration was employed, consisting of a saturated calomel reference electrode (E° = +0.242 V vs. ENH), the painted sample as the working electrode (with an exposed area of 19.64 cm^2^), and a platinum counter electrode. The measurement was taken after 30 min of the sample being at the corrosion potential.

## 3. Results and Discussion

### 3.1. Characterization of the Test Atmosphere

Table 4 presents the annual average values of the meteorological variables recorded during the exposure period. These variables include temperature (T), relative humidity (RH), amount of rainfall, wind speed, and solar radiation. It is important to mention that in the evaluation area during the study years, there is a marked difference between the maximum and minimum temperatures (see Table S1), a condition that is not registered in the rest of the country.

Table 5 displays the assessment of the atmospheric aggressiveness category based on the annual averages of chloride deposition, sulfur dioxide deposition, and time of wetness, following the guidelines outlined in ISO 9223 [23]. The evaluation results indicate a categorization of C3, which corresponds to medium aggressiveness. Considering the classification, this site can be primarily characterized as a marine environment since the pollution from sulfur dioxide (SO_2_) is insignificant, indicating a classification of P0 (negligible SO_2_ pollution).

### 3.2. Corrosion of Carbon Steel

**Figure 3:**
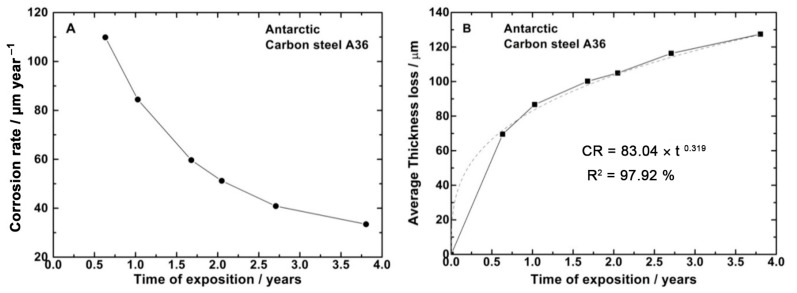
Corrosion rate (**A**) and thickness loss (**B**) of A36 steel as a function of exposure time.

Figure 3A,B illustrate the average variation in corrosion rate and steel thickness loss, respectively, as a function of exposure time. The overall trend indicates a decrease in corrosion rate with increasing exposure time, which is influenced by the protective nature of the corrosion product formed on the steel (including its morphology and microstructure) as well as the environmental characteristics.

Furthermore, a visible increase in the macroscopic corrosion product formation on the steel is observed with longer exposure durations. According to ISO 9226 [25], the corrosivity classification of A-36 carbon steel after one year of exposure in Antarctica falls within category C5, denoting very high corrosivity (85.64 µm/year). However, this value is near the upper limit of C4 (high corrosivity) and the lower limit of C5 corrosivity.

Previous research conducted under the MICAT project examined steel exposed at Marsh Station, located near the coast. In that study, the corrosion rate after one year of exposure was measured to be 24.1 µm/year (corrosivity category C2). The environmental conditions, such as temperature, relative humidity, and time of wetness, were similar to those of the present study. However, data on ambient chloride concentration and wind speed are unavailable, and the annual rainfall was lower [2,3].

The average ambient chloride content recorded over the 45-month study period was 29.87 mg/m^2^ per day (category S1), while the sulfur dioxide content (SO_2_) was 2.65 mg/m^2^ per day (category P0). These values indicate a marine atmosphere with low chloride content and negligible sulfur dioxide content. In such conditions, the water layer between the metal and ice may contain higher chloride concentrations, thereby delaying the freezing process and activating the corrosion of the material.

Comparing the corrosion rate obtained during the 2014–2015 period to the first year of the 1988–1994 period [2,3], it is observed that the corrosion rate in the later period is 3.5 times higher. This difference can be attributed to the structure and morphology of the corrosion product, which allows for the maintenance of an active water layer at both the metal/corrosion product interface and the corrosion product/ice interface. Electrochemical measurements conducted by Rosales et al. demonstrated the presence of an electrolyte layer at the metal/ice interface [30].

Consider one of the long-term prediction models given by the potential function *CR* = *A*·*t^n^*, which can be rewritten into the bi-logarithmic model:

log_10_*CR* = log_10_*A* + *n*·log_10_*t* + ε (1)

where *CR* is the magnitude of corrosion (loss of thickness) of the metal at *t* years; *t* is the exposure time of the metal, in years; *A* is the measure of corrosion (loss of thickness) at the first year of exposure (*t* = 1); and *n* is an indicator parameter of the physical–chemical behavior of the corrosion layer and its interactions with the atmosphere [31,32,33]. This gives the relationship as:

(2) $$CR=83.040\times t^{0.319}{\;R}^{2}=97.92\;\%$$

for which, under the given conditions, the variable n exhibits a value less than 0.5, indicating that the corrosion mechanism is controlled by diffusion through the corrosion product formed on the steel surface.

**Figure 4:**
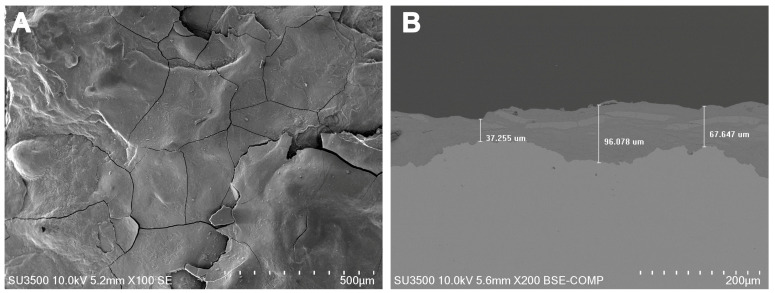
SEM of the carbon steel corrosion product after 45 months of exposure: (**A**) superficial, (**B**) cross-section.

Figure 4 shows the microphotographs obtained using SEM for the steel corrosion product after 45 months of exposure. In Figure 4A, the formation of flat and smooth layers can be observed, resulting from the deposition of an ice layer on the material. These layers exhibit cracks, typically parallel to the steel surface, due to the accumulation of soluble salts and impurities between the sublayers, as mentioned earlier. These cracks can undergo state changes caused by temperature variations, particularly temperatures below 0 °C, leading to the rupture of the oxide layer [3,34].

Figure 4B provides a cross-section view of the corroded steel, confirming the layered structure of the corrosion product with cracks present. The internal part appears more compact, while the external part shows a slight tendency to lose adherence. The thickness of the steel corrosion product, as depicted in Figure 4B, ranges between 37 and 96 µm.

The composition of the corrosion product, as determined by XRD analysis, revealed a mixture predominantly composed of lepidocrocite (γ-FeOOH) as the main component, along with goethite (α-FeOOH) as the secondary component. These findings align with results reported by other authors [35,36,37], indicating consistency in the composition of the corrosion product.

### 3.3. Evaluation of Coating Deterioration

After a 45-month exposure period in Antarctica, the painted samples did not exhibit a significant change in paint layer thickness. The recorded thickness variations were within the measurement deviation range, indicating minimal alteration. Visual inspections of the three paint systems were conducted following ISO 4628-2 [38] standards, and the results are presented in Table 6.

According to ISO 4628-2, which considers blister density and size, very few blisters were observed in all three paint systems. The blisters had low density and were negligible in size when observed with the naked eye. Therefore, the three paint systems are classified as 2(S2) in terms of blistering.

The surface rust of the evaluated systems, categorized according to ISO 4628-3 [39], was determined to be Ri 0. No appreciable oxides were observed on the paint surface.

Furthermore, according to ISO 4628-4 [40], only a minimal number of cracks was observed in all three paint systems. These cracks were hardly significant and visible at magnifications of up to 10x.

As for flaking, assessed following ISO 4628-5 [41], the amount and density of flaking were low for the M1 and M2 systems—i.e., top coat of polyurethane—and were classified as 1(S0)a. The M3 system, which had an epoxy top coat, exhibited slightly more noticeable flaking, resulting in a classification of 1(S1)a. It is important to note that the observed defects were limited to the surface level, and no damage was observed beyond the top coat in any of the three systems.

Table 7 presents the adhesion measurements of the three paint systems under initial conditions and after 45 months of exposure to the Antarctic environment. Coatings exposed to atmospheric corrosion in this region face unique challenges due to the harsh climatic and environmental conditions present. The corrosive nature of the Antarctic environment is intensified by factors such as low temperatures, high humidity, exposure to snow and ice, strong winds, and ultraviolet radiation.

In light of these conditions, it was observed that the tensile adhesion values of the paints decreased after exposure to extreme climatic conditions. However, it is important to note that these values remained above 2.5 MPa. According to ISO 12944-6 [42], this level of adhesion is crucial in preventing moisture penetration and the onset of corrosion beneath the coating.

The observed failures were mainly attributed to cohesion problems in the top layer of the M2 system, which utilizes a zinc-rich coating as a primer layer. These results confirm the strong adhesion of this coating to the substrate and subsequent layers of the system. On the other hand, the M1 system, which employs a self-primer, exhibited adhesion failures with the metal. This could be attributed to the lower thickness of the coating compared to that of the M3 paint system, which also utilizes the same self-primer. However, these coatings are susceptible to degradation in environments characterized by high humidity, salt content, oxidation, and exposure to solar radiation. Such degradation leads to a decrease in physicochemical and mechanical properties, resulting in coating deterioration [43,44,45]. Therefore, it is crucial to predict the lifespan of these coatings by gaining a deeper understanding of the phenomena occurring within the polymer, especially when subjected to “physical aging.” This phenomenon refers to structural relaxations in the polymer chains occurring below the glass transition temperature (Tg) as a means to reach an equilibrium state. When polymers are cured above Tg and subsequently cooled below it, the polymeric network remains in a state of disequilibrium as the polymeric chains do not have enough time during cooling to achieve equilibrium [46]. Consequently, there is an increase in volume, enthalpy, and entropy values, indicating that the polymeric network tends to return to an equilibrium state during its life cycle, ultimately leading to a decrease in free volume. Researchers have associated this phenomenon with water absorption in coatings, recognizing it as a significant issue since water penetration is the initial stage of the degradation process [45].

In summary, the results demonstrated that the evaluated paint systems maintain good adhesion and resistance to delamination even after prolonged exposure to Antarctic meteorochemical conditions. However, variations in the performance of the systems were observed, particularly regarding the cohesion of the top coat and adhesion to the substrate, which are linked to the specific characteristics of each paint system.

Table 8 presents the contact angle results of the painted specimens before and after 45 months of exposure in Antarctica; see Figure S2. Significant changes in contact angle were observed for the aliphatic acrylic polyurethane (M1) and aliphatic polyurethane (M2) top coats after the 45-month exposure, decreasing from 75.1° to 67.8° and from 77.9° to 60.3°, respectively. These changes are attributed to the degradation and leaching of coating components caused by exposure to UV radiation, snow and ice, strong winds, and salt spray [47]. These environmental factors are known to impact the surface structure of the coating, resulting in a decrease in contact angle [48].

In contrast, the contact angle of the epoxy surface (M3) increased from 116.5° to 84.1°; see Figure S2E,F. This noticeable change can be explained by the exposure of aluminosilicate type charge due to UV degradation of the resin. Previous studies have shown that UV radiation exposure can lead to changes in the surface of polymers, resulting in the exposure of charges such as aluminosilicates. These groups may have hydrophobic properties that could have increased the surface wettability [49], which is not reflected in an increase in the contact angle, but rather in the dispersive components presented by the M3 samples [50]; this was not observed in samples M2 and M1.

The results obtained from the abrasion test are shown in Figure S3 and presented in Table 9. These show that the unexposed coatings present abrasion index values higher than those of the reference established in the ASTM D4060 Standard for polyurethane systems (50 mg/1000 cycles) and slightly higher for the epoxy finishing system (120 mg/1000 cycles). These values increase for the three coatings after a period of exposure of 45 months to the atmospheric conditions of the Antarctic.

The M1 coating system—consisting of an aliphatic acrylic polyurethane top coat—exhibited better abrasion resistance compared to the aliphatic polyurethane (M2) and epoxy (M3) systems. The increase in abrasion rate was 38, 70, and 78 mg/1000 cycles for the M1, M2, and M3 samples, respectively. This increase in abrasion index or decrease in abrasion resistance is expected due to the degradation caused by UV radiation, chalking, blistering, loss of gloss, and possibly the appearance of surface microcracks in the top layer of the analyzed systems. It is important to note that the system with an epoxy top coat was the most affected, suggesting a possible degradation of the resin used in that system. The increase in the abrasion index was lower for the M1 system, as expected for systems containing an aliphatic acrylic polyurethane top coat under similar conditions. On the other hand, the M2 system showed a considerable increase compared to M1, which could be attributed to the greater flexibility of the aliphatic polyurethane top layer compared to the aliphatic acrylic polyurethane. This increased flexibility, when exposed to repeated cycles of contraction and expansion due to ice and thaw in Antarctica, may lead to microcrack formation and coating degradation. This behavior is supported by the results of gloss loss of the coatings presented in Table 9, where the increase in the abrasion index correlates with the loss of gloss.

Table 10 provides the results of gloss loss (expressed in gloss units, GU) and color shift (expressed in color tolerance system units, CMC) after 45 months of exposure to atmospheric conditions in Antarctica. The polyurethane coatings in the top coat show different behaviors (Figure S4, Table 10). The M2 system, which utilizes aliphatic polyurethane, exhibits the highest gloss loss (53.9%) and the lowest color change (CMC 1.3). This suggests that this type of coating is more susceptible to gloss degradation, possibly due to leaching of coating components and degradation caused by ultraviolet (UV) radiation.

On the other hand, the aliphatic acrylic polyurethane of the M1 system demonstrates moderate gloss loss (6.8%) and a higher CMC (5.73). This indicates that this top coat may be more prone to color fading, possibly due to leaching of components and degradation of pigment types caused by UV radiation. These factors can influence changes in the color of the coated surface [51,52].

**Figure 5:**
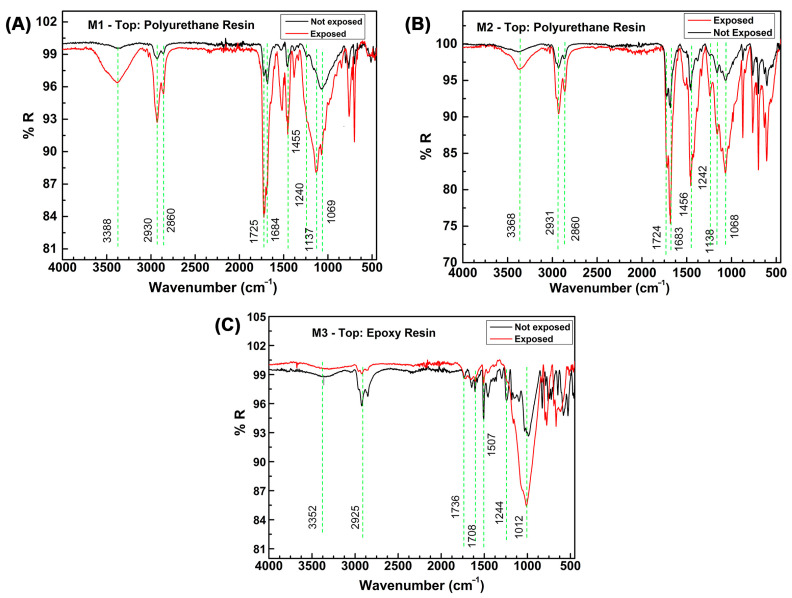
FTIR-ATR spectra of the control and exposed samples at 45 months: (**A**) M1; (**B**) M2; and (**C**) M3.

In the case of the epoxy coating, the highest gloss loss (88.8%) and moderate CMC (4.13) are observed. These results indicate that this type of coating is highly susceptible to degradation caused by UV radiation and extreme atmospheric conditions, likely due to its chemical composition and molecular structure [53,54,55]. Degradation of the resin in the surface layer may lead to an imbalance in the resin-to-pigment ratio, resulting in a decrease in gloss as less light is reflected on the surface. This phenomenon is supported by infrared analysis performed on the top layer of the epoxy resin, where significant variations in the vibrational bands corresponding to characteristic epoxy groups are observed (Figure 5).

In summary, the results highlight the different behaviors of polyurethane and epoxy coatings in terms of gloss loss and color fading after exposure to Antarctic atmospheric conditions. These findings are important for understanding the durability and performance of coatings exposed to extreme environments, providing valuable insights for the development of corrosion protection strategies in such conditions.

Figure 5 illustrates the FTIR-ATR spectra of the polyurethane (M1 and M2) and epoxy (M3) top coats on both unexposed and exposed painted samples in Antarctica. The spectra clearly demonstrate a discernible difference in the % reflectance of the studied resins between the initial samples and those subjected to 45 months of exposure in the cold climate with high UV radiation found in Chilean Antarctica.

During the evaluation period of the coatings, moderate UVI indices in the range of 3 to 5 were observed at the test site. Although these values are considered moderate, it is important to note that the UVI is calculated specifically from arithmetic ultraviolet radiation in the range 250 to 400 nm, which encompasses the near and intermediate regions of UV radiation, i.e., UV-A and UV-B, respectively. However, the highest energy far region, known as UV-C (200–280 nm), is not taken into account due to its predominant absorption by the stratospheric ozone layer [56].

It is relevant to mention that, during the evaluation period, values below the threshold of 220 D.U. (Dobson units) were recorded for the stratospheric ozone column. In addition, a longer duration and extension of the ozone hole was observed, with an average ozone mass deficit of approximately 30 megatonnes, the highest value recorded in recent decades. These results indicate a significant decrease in the stratospheric ozone concentration [57,58,59,60]. Consequently, it is estimated that, due to this decrease in the stratospheric ozone layer, the higher energy UV radiation is not entirely absorbed and reaches the surface of the samples studied, which causes more significant degradation of the resins used in the coatings.

**Figure 6:**
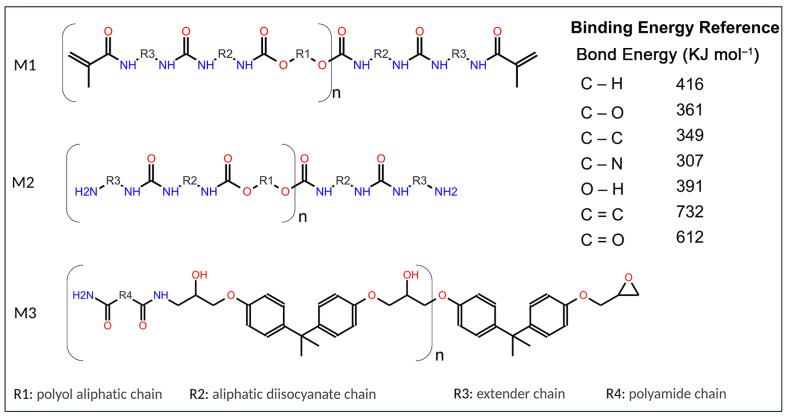
Reference chemical structures for the polymers used in the topcoat resin. M1, Aliphatic acrylic polyurethane; M2, Aliphatic polyurethane; M3, Epoxy.

One of the main causes of polymer degradation lies in its intrinsic chemical composition. Within this composition, it is crucial to highlight the presence of specific chemical groups, which can be found in the main chain or the branches, depending on whether the polymer has a linear or branched structure. As is known, any chemical reaction that affects an organic compound leads to the breaking of covalent bonds. Therefore, the value of the binding energies provides information about the stability of a particular bond or chemical group (Figure 6). However, in the case of polymers, this factor is not the only determinant, and consequently, the chemical groups or bonds involved in these reactions may exhibit a different chemical reactivity than that of simple molecules [61].

In addition, there are other factors of a structural nature, such as spatial conformation and glass transition temperature, which influence the stability or changes in the polymer matrix [62]. According to the above, crystallinity hinders the diffusion of the agents responsible for the chemical transformation of the polymers and access to their corresponding reactive groups. Therefore, for a given chemical composition, crystalline or semi-crystalline polymers will be more stable than those with an amorphous structure.

According to the results presented in Figure 5, in the case of the polyurethane resins (M1 and M2), a decrease in % reflectance is observed at 3388 cm^−1^ and 3368 cm^−1^, respectively, indicating the breakdown of secondary amine bonds. The bands around 2930 cm^−1^ and 2860 cm^−1^ suggest a decrease in acyclic C-H bonds. The reduction in the bands located near 1725 cm^−1^ and 1684 cm^−1^ is attributed to changes in the carbonyl group of the amide, indicating the degradation of the polyurethane groups. Additionally, a decrease in the bands at 1455 cm^−1^ and 1240 cm^−1^ indicates a reduction in C-H and C-O bonds, respectively. The emergence of the stretching alcohol C-O band at 1068 cm^−1^, along with the decrease in the stretching broad ester C-O band at 1137 cm^−1^, are likely associated with the hydrolysis reaction of the ester group within the polyurethane coating. Hence, it is suggested that the chemical changes in the polyurethane coating, particularly M2 (aliphatic), following atmospheric exposure, could involve ester group hydrolysis and the formation of hydroxyl groups [45].

Regarding the specimens coated with an epoxy resin finish (M3), a decrease in % reflectance is also observed compared to the initial unexposed sample and those exposed to the atmosphere. Studies have indicated that the -CH group of the amino group is prone to oxidation, resulting in the formation of new amide groups [9,63]. Additionally, the C-O stretching of aldehydes or ketones at 1708–1736 cm^−1^ and the decrease in the CN band intensity at 1244 cm^−1^ suggest that the chemical structure of the epoxy coating has been affected by aging due to atmospheric exposure. The reduction in band intensity at 1012 cm^−1^ indicates the breakage of ether functional group bonds, which are predominant in epoxy resins. Consequently, M3 exhibits a significant gloss loss of 89% and resin degradation, resulting in chalking and variation in the pigment-to-binder ratio [64].

Additionally, UV degradation of resins contributes to chalking, delamination, and discoloration, and adversely affects the gloss, hardness, and surface roughness of coatings. Although only 5% of the total solar UV radiation reaches the Earth’s surface (280–400 nm), its high energy can easily initiate the formation of free radicals, leading to chain scission and subsequent oxidative reactions of polymer chains [53,54].

While it is challenging to determine bond degradation solely based on the decrease in intensity of characteristic bands, consistent concentrations and/or sample amounts in each test may be facilitated by FTIR-ATR analysis. Thus, in a comparative analysis between the two resins, the epoxy resin exhibited a more substantial decrease in characteristic bands, indicating greater degradation, which aligns with other reported physical tests such as abrasion, loss of brightness, and color.

**Figure 7:**
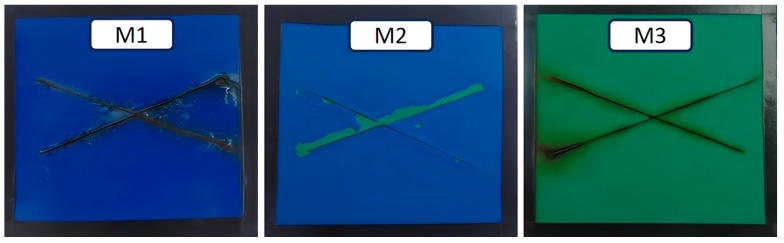
Surface appearance of the Evans X-Cut in the painted samples after 45 months of exposure.

Furthermore, Figure 7 displays the surface appearance of the Evans X-Cut samples after 45 months of exposure in Antarctica. When a cut is made on the paint, exposing the bare metal to the environment, the corrosion process occurs, influenced by the aggressiveness of the surroundings. The exposed area behaves anodically, while the painted area behaves in a cathodic manner, thereby accelerating the corrosion process of the bare metal. The initiation and propagation of the corrosion process on the underlying steel of the paint system are associated with the adhesion capacity of the primer. In this case, the presence of rust is observed in the groove of samples M1 and M3, both of which have an epoxy primer, along with blistering of the paint surrounding the rusted areas. On the other hand, sample M2, which has an epoxy rich in zinc as a primer, does not exhibit rust formation in the groove, as expected due to the sacrificial anode action of zinc. However, sample M2 experiences partial loss of the polyurethane top layer (aliphatic) around the groove, a phenomenon that does not occur to the same extent in sample M1, which has a polyurethane top layer composed of an acrylic–aliphatic mixture.

Aliphatic polyurethane paints generally possess good plasticity, exhibiting elasticity and flexibility. They have the capacity to adapt to dimensional changes and minor surface deformations, enabling them to maintain their integrity and resistance over time. This inherent plasticity provides several advantages, including resistance against cracking and fissuring caused by structural movements, vibrations, or moderate thermal changes. Moreover, their flexibility allows them to endure impacts, abrasions, and deformations without compromising their protective properties or appearance.

However, in extremely low-temperature conditions such as those found in Antarctica, the plasticity of aliphatic polyurethane paints can be reduced, increasing the risk of fractures, cracks, and poor adhesion to the epoxy interlayer. This, in turn, can lead to delamination or detachment of the polyurethane layer. Strong winds in the Antarctic environment can also damage the paint surface and impose additional mechanical stresses on the structure, further contributing to cracking or detachment of the top coat.

Regarding the acrylic–aliphatic polyurethane system, the presence of acrylic components with double bonds results in areas with greater mechanical and chemical resistance to degradation. As a result, this mixture exhibits improved cohesion and better resistance to changes in flexibility of the resins due to temperature variations, particularly in extreme cases.

**Figure 8:**
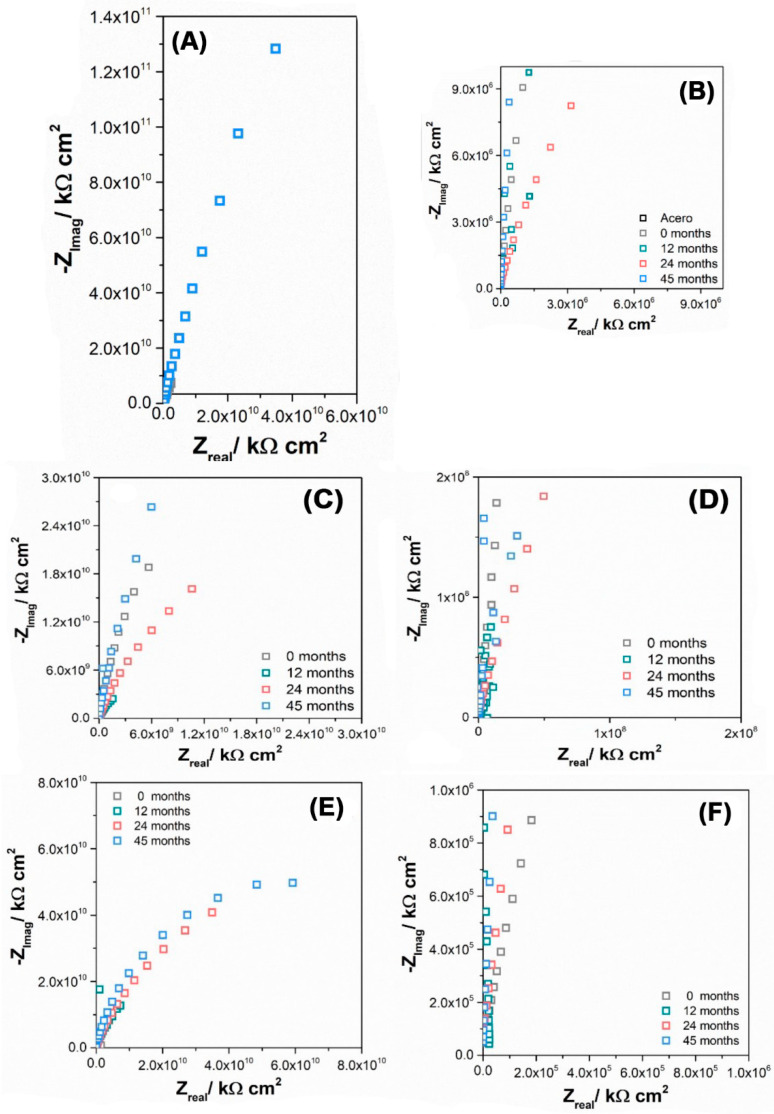
Impedance response of M1 paint system at different exposure times to the environment, measured in 0.1 M Na_2_SO_4_ solution at E = OCP: (**A**) Nyquist plot and (**B**) magnification of Nyquist plot for M1; (**C**) Nyquist plot and (**D**) magnification of Nyquist plot for M2 and (**E**) Nyquist plot and (**F**) magnification of Nyquist plot for M3.

Figure 8 depicts the Nyquist plot response of the paint schemes exposed to the environment at different studied exposure times, conducted in a 0.1 M Na_2_SO_4_ solution.

All the samples exhibit a non-ideal capacitive behavior across the entire frequency range, which can be attributed to the distribution of current and potential over the surface [65]. This behavior is reflected in the constant phase element (CPE) parameters, α and Q_CPE_, as shown in Table 11.

From the analysis of the Bode diagrams, the modulus values at 0.1 Hz were extracted (see Table 11). These values indicate that none of the three paint systems show a significant loss of protection over time, as none of the systems have moduli close to that of bare steel (measured modulus of 279.6 Ω cm^2^). The table shows that all the paint systems have an average modulus value of |Z| ≥ 10^10^ Ω cm^2^. Additionally, the values of α suggest that the paints maintain their homogeneity over time [66]. On the other hand, we can consider the proposal of Lee C. et al. [66], where the classification of the performance of the coating can be carried out considering the anticipated value of the log |Z|0.1 and where it is established that the coating can be classified as follows:

(i) Excellent protection if log |Z|0.1 is >10;
(ii) Good protection if log |Z|0.1 is >8;
(iii) Poor protection if log |Z|0.1 is <4.

From here, it can be said that all paint schemes have excellent protection even after 45 days of exposure.

When comparing the different paint schemes, it can be observed that the M1 scheme exhibits a more consistent behavior over time, as its modulus remains relatively stable and the decrease in Q_CPE_ is not drastic compared to the M2 scheme. On the other hand, the M2 scheme does not show a clear trend in modulus variation, but Q_CPE_ decreases after 45 months. This suggests that the coating loses its protective properties over time, possibly due to a loss in film homogeneity, which aligns with previous observations regarding changes in tensile strength and adhesion to the intermediate paint layer.

## 4. Conclusions

The carbon steel used in the study was classified to corrosivity category C5, with a corrosion rate of 85.64 µm yr^−1^ after one year of exposure in Chilean Antarctic Territory. The corrosion kinetics of the steel under the study conditions are described by the function *CR*_cs_ = 83.04 × *t*^0.319^ (R^2^ = 97.92%).

The results obtained after subjecting the three most prevalent industrial paint systems in Chile to adverse weather conditions, such as those in Antarctica (high temperature fluctuations, winds, and UV), revealed a good performance in general terms, demonstrating adequate adhesion and resistance to delamination, as well as the absence of surface oxide. However, some aging effects were observed, such as loss of gloss, increased abrasion index, and decreased percentage reflectance.

The coating system, which included an aliphatic acrylic polyurethane top coat (M1), demonstrated better performance in terms of its mechanical properties compared to systems that had an aliphatic polyurethane (M2) or epoxy (M3) top coat. However, in cases where the surface coating suffered some mechanical damage that reached the base metal, it was observed that the system with a primer rich in zinc (M2) achieved better performance by avoiding a significant growth in the oxide layer, due to the galvanic effect of zinc. In addition, the electrochemical impedance analysis yielded encouraging results since it indicated that the protective nature of the coatings in the three schemes was maintained after 45 months of exposure to the environment.

## Figures and Tables

**Table 1:** Chemical composition of A-36 steel (%).

**C**	**Si**	**Mn**	**P**	**S**	**Cr**	**Ni**	**Mo**	**Al**
0.185	0.136	0.388	0.014	0.008	0.032	0.020	0.002	0.037
**Cu**	**Co**	**Ti**	**Nb**	**V**	**W**	**Sn**	**B**	**Fe**
0.012	0.008	0.001	<0.001	0.002	0.009	0.012	0.0008	All else

**Table 2:** High-durability paint systems.

System (Corrosivity)	Primer	Intermediate Coat	Top Coat	Average Total Thickness (µm)
M1	Self-priming epoxy	Enamel Epoxy	Polyurethane Acrylic–aliphatic (blue color)	259 ± 30
M2	Zn-rich epoxy	Enamel Epoxy	Polyurethane aliphatic (blue color)	306 ± 37
M3	Self-priming epoxy	Enamel Epoxy	Epoxy (green color)	390 ± 26

**Table 3:** Top layer properties in paint systems tested.

M1	M2	M3
Polyurethane aliphatic acrylic	Polyurethane aliphatic	Polyamide epoxy
High gloss and chemical resistance	Brilliant	Semi-gloss
Solids 65 +/−3%	Solids 67 +/−1%	High solids 80 +/−1%
Good color retention and high UV fastness	Good gloss and color retention	Generates a thick, durable, tenacious coating

**Table 4:** Average meteorological variables at the Antarctica station.

Exposure Period (Years)	Temperature (°C)	RH (%)	V. Wind (m/s)	Cumulative Solar Radiation (kW/m^2^)	Cumulative Precipitation (mm)
1 (2014–2015)	−3.0	88.0	17.4	1368.3	867.8
2 (2015–2016)	−3.0	89.0	17.9	1687.9	843.0
3 (2016–2017)	−1.6	89.0	22.6	1426.5	879.2
4 (2017–2018)	−2.5	88.5	19.2	1389.2	858.4

**Table 5:** Average levels of chemical agents; time of wetness and environmental aggressiveness category at the Antarctica station.

Exposure Period (Years)	Chloride Deposits (mg/m^2^ Day)	SO_2_ Deposits (mg/m^2^día)	TOW (Hours/Year)	Corrosivity Category (ISO 9223)
1 (2014–2015)	22.536	3.365	3918.8	S1P0τ4/C3
2 (2015–2016)	35.483	2.321	4520.4	S1P0τ4/C3
3 (2016–2017)	28.982	2.076	4921.2	S1P0τ4/C3
4 (2017–2018)	32.479	2.856	4765.2	S1P0τ4/C3

**Table 6:** Visual inspection classifications of paint systems at the Antarctica Station after exposure for 45 months.

	Quantity (Density) at 45 Months of Exposure			
Paint System	Blistering (ISO 4628-2)	Rusting (ISO 4628-3)	Cracking (ISO 4628-4)	Flaking (ISO 4628-5)
M1	2(S2)	Ri 0	1(S1)a	1(S0)a
M2	2(S2)	Ri 0	1(S1)a	1(S0)a
M3	2(S2)	Ri 0	1(S1)a	1(S1)a

**Table 7:** Adhesion of paint systems after exposure during 45 months of exposure at the Antarctica Station.

Initial				45 Months of Exposure			
Paint System	Tensile Strength (MPa)	Type of Failure/Layer	% Failure	Paint System	Tensile Strength (MPa)	Type of Failure/Layer	% Failure
M1	9.9	- Cohesion A - Adhesion B/C - Adherence	50 20 30	M1	9.6	- Adhesion M/A	100
M2	11.7	- Adhesion M/A - Adherence P	80 20	M2	7.5	- Cohesion C	100
M3	8.9	- Cohesion C - Adhesion B/C	90 10	M3	7.7	- Cohesion C - Adhesion B/C	90 10

M = Metallic substrate; A = Primer; B = Intermediate; C = Top; P = Glue.

**Table 8:** Characteristics of the types of samples in terms of physical properties: free energy (γ) and polar ($\gamma_{s}^{p}$) and dispersive ($\gamma_{s}^{d}$) components obtained by static contact angle measurements (θ) in water and diiodometane.

System	θ_Water_ (°)	θ_diiodometane_ (°)	γ (Mn/m)	$\gamma_{s}^{d}\;(\mathrm{Mn}/m)$	$\gamma_{s}^{p}\;(\mathrm{Mn}/m)$
Initial					
M1	75.1 ± 4.2	47.3 ± 0.17	44.77 ± 5.53	30.87 ± 0.65	13.89 ± 4.88
M2	77.9 ± 6.4	67.9 ± 0.14	47.23 ± 9.32	31.25 ± 1.02	15.98 ± 8.29
M3	116.5 ± 7.7	67.9 ± 0.14	40.49 ± 0.32	45.96 ± 0.49	35.02 ± 0.33
45 Months					
M1	67.8 ± 0.3	55.58 ± 0.24	47.25 ± 7.50	29.00 ± 0.74	18.25 ± 6.76
M2	60.3 ± 4.7	58.32 ± 0.32	51.00 ± 8.69	25.89 ± 0.74	25.11 ± 7.95
M3	84.1 ± 5.8	43.56 ± 0.38	41.33 ± 5.33	35.52 ± 0.96	5.81 ± 4.37

**Table 9:** Abrasion resistance of paint systems after exposure for 45 months at the Antarctica Station.

Abrasion Index (mg/1000 Cycles)		
Paint System	Initial	45 Months
M1	102 ± 20	140 ± 4
M2	150 ± 35	220 ± 20
M3	142 ± 21	260 ± 20

**Table 10:** Brightness and color measurements of paint schemes after exposure during 45 months at the Antarctica Station.

Paint System	Brightness (GU)		Color (CMC)
	Initial	45 Months	45 Months
M1	86.7	80.8	5.73
M2	65.5	30.2	1.30
M3	11.6	1.3	4.13

**Table 11:** Impedance parameters of steel probes at different exposure times.

Time (Months)	Probe	|Z|_0.1Hz_ × 10^10^/Ω cm^2^	$-\alpha_{HF}$	Q_CPE_/Fs^(α−1)^cm^−2^ × 10^−11^
0	M1	2.70	0.931	7.45
12		1.24	0.981	3.75
24		0.13	0.953	5.63
45		2.53	0.975	4.37
0	M2	1.05	0.923	13.9
12		0.31	0.929	12.4
24		0.75	0.954	10.6
45		1.58	0.922	7.11
0	M3	3.60	n.d	n.d
12		1.48	0.991	3.39
24		1.18	0.963	5.35
45		1.94	0.974	4.05

## Data Availability

The raw/processed data required to reproduce these findings cannot be shared at this time due to technical or time limitations.

## Supplementary Materials

The following supporting information can be downloaded at: https://www.mdpi.com/article/10.3390/ma16165713/s1, Figure S1: Painted and unpainted samples were exposed at the monitoring station; Figure S2: Contact angles of coating systems before and after 45 days of exposure. (A) M1 before; (B) M1 after; (C) M2 before; (D) M2 after; (E) M3 before and (F) M3 after; Figure S3: Abrasion images of the coating systems before and after 45 days of exposure; Figure S4: Samples of coating systems before and after exposure for 45 months in Antarctica; Table S1: Summary of temperatures and precipitations, by meteorological station, 2013–2017 President Frei Antarctic base (Lat. 62 25S. Long. 5853`O. Alt. 10 msnm).
