# Supplementary material for: Performance of Anticorrosive Paint Systems for Carbon Steel in the Antarctic Marine Environment
Source: Materials (Basel). 2023 Aug 21;16(16):5713. doi: 10.3390/ma16165713 (PMC10456802; doi:10.3390/ma16165713)
Supplement: Supplementary file 1 [file materials-16-05713-s001.zip › materials-2509677-supplementary.pdf]

# Performance of Anticorrosive Paint Systems for Carbon Steel in the Antarctic Marine Environment

## Supplementary Materials.

**Rosa Vera [1,*], Margarita Bagnara [1], Rodrigo Henríquez [1], Lisa Muñoz [1], Paula Rojas [2] and Andrés Díaz-Gómez [1,*]**

[1] Instituto de Química, Facultad de Ciencias, Pontificia Universidad Católica de Valparaíso, Av. Universidad 330, Placilla (Curauma), Valparaíso 2373223, Chile; margarita.bagnara@pucv.cl (M.B.); rodrigo.henriquez@pucv.cl (R.H.); lisa.munoz@pucv.cl (L.M.)
[2] Facultad de Ingeniería y Ciencias, Universidad Adolfo Ibáñez, Diagonal Las Torres 2640, Santiago 7941169, Chile; paula.rojas.s@uai.cl
* Correspondence: rosa.vera@pucv.cl (R.V.); andresdiaz.qind@gmail.com (A.D.-G.)

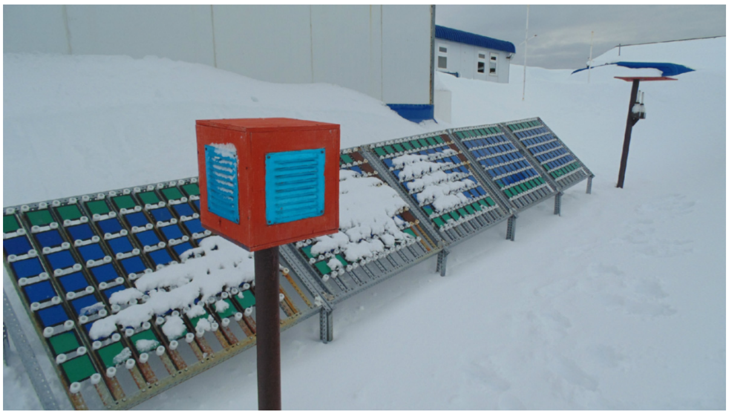

**Figure S1.** Painted and unpainted samples were exposed at the monitoring station.

**Table S1.** Summary of temperatures and precipitations, by meteorological station, 2013-2017 President Frei Antarctic base (Lat. 62 25S. Long. 5853`O. Alt. 10 msnm)

| AÑO Y MES | Temperatura °C | | | Agua caída (mm.) |
|---|---|---|---|---|
| | Máxima absoluta | Mínima absoluta | Media | |
| **2013** | 5,5 | -19,8 | -2,6 | 302,1 |
| **2014** | 4,0 | -17,4 | -2,5 | 1064,4 |
| **2015** | 7,0 | -19,6 | -3,0 | 817,5 |
| **2016** | 5,8 | -19,6 | -1,8 | 1035,6 |
| **2017** | 6,0 | -20,1 | -2,1 | 1336,4 |
| **2017** | | | | |
| Enero | 4,9 | -1,9 | 1,2 | 33,6 |
| Febrero | 5,2 | -4,6 | 1,6 | 68,4 |
| Marzo | 6,0 | -6,7 | 0,9 | 57,1 |
| Abril | 3,7 | -13,7 | -1,9 | 87,4 |
| Mayo | 4,2 | -12,1 | -2,3 | 145,5 |
| Junio | 1,3 | -19,4 | -5,9 | 173,3 |
| Julio | 1,9 | -20,1 | -4,2 | 126,8 |
| Agosto | 1,1 | -17,9 | -5,6 | 122,5 |
| Septiembre | 1,6 | -14,4 | -4,4 | 96,2 |
| Octubre | 2,9 | -8,7 | -3,1 | 264,2 |
| Noviembre | 2,3 | -8,3 | -1,6 | 94,7 |
| Diciembre | 5,3 | -4,7 | 0,1 | 66,7 |
| **Año normal(1** | | | **-2,3** | **797,2** |

**(1)** Valores de año normal calculados para el período 1961-1990.
Fuente: Dirección Meteorológica de Chile.

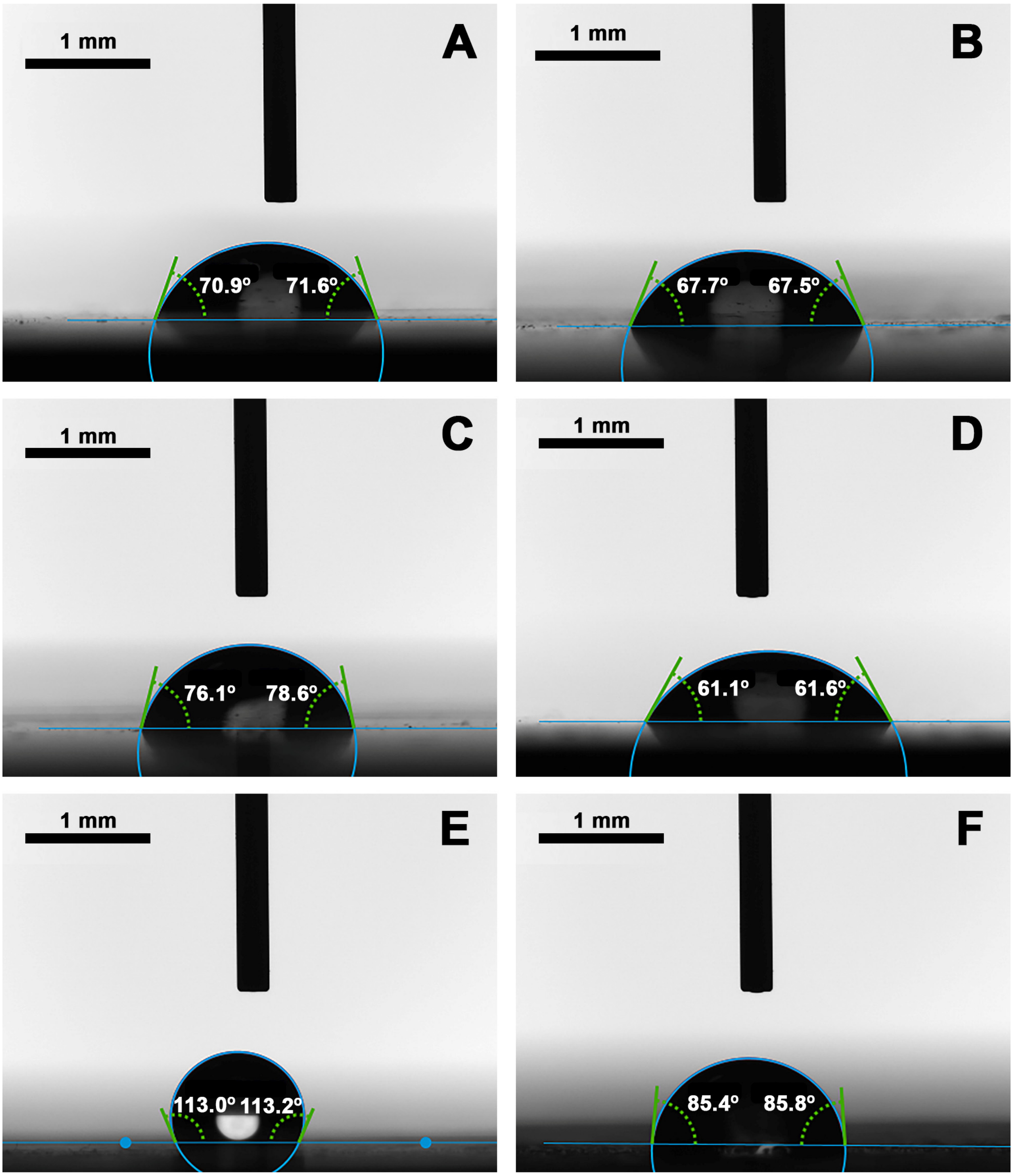


**Figure S2.** Contact angles of coating systems before and after 45 days of exposure. A) M1 before; B) M1 after; C) M2 before; D) M2 after; E) M3 before and F) M3 after.

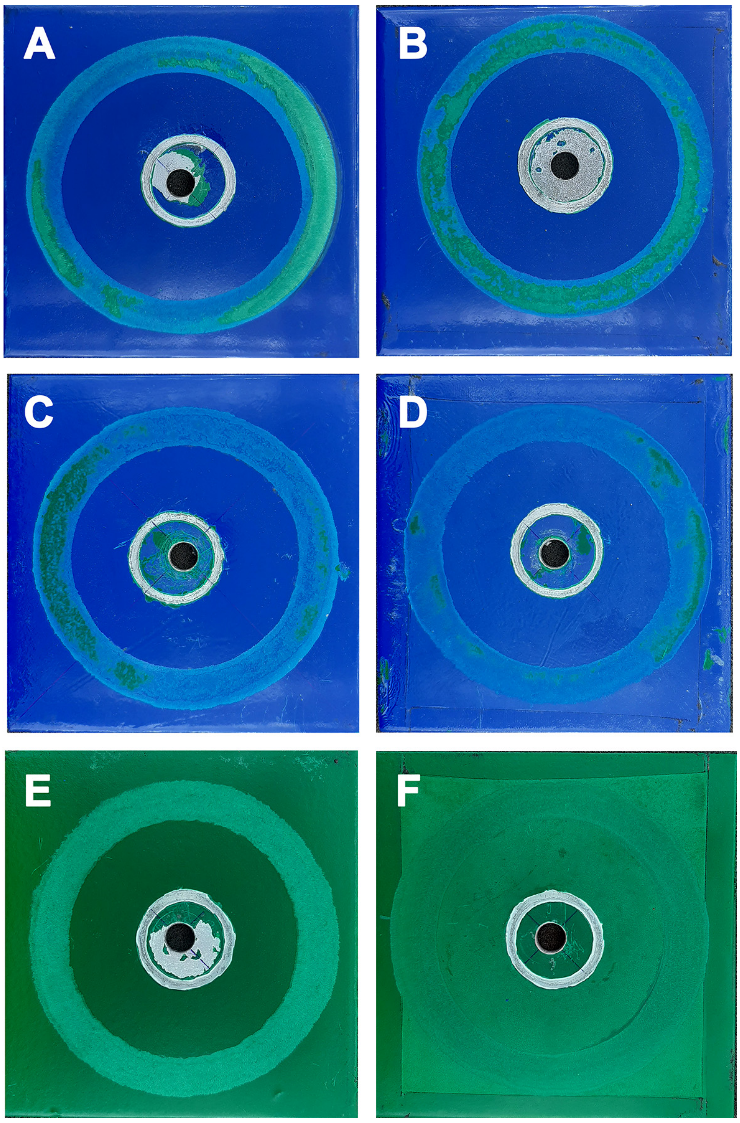


**Figure S3.** Abrasion images of the coating systems before and after 45 days of exposure. (A-B) M1, (C-D) M2, and (E-F) M3.

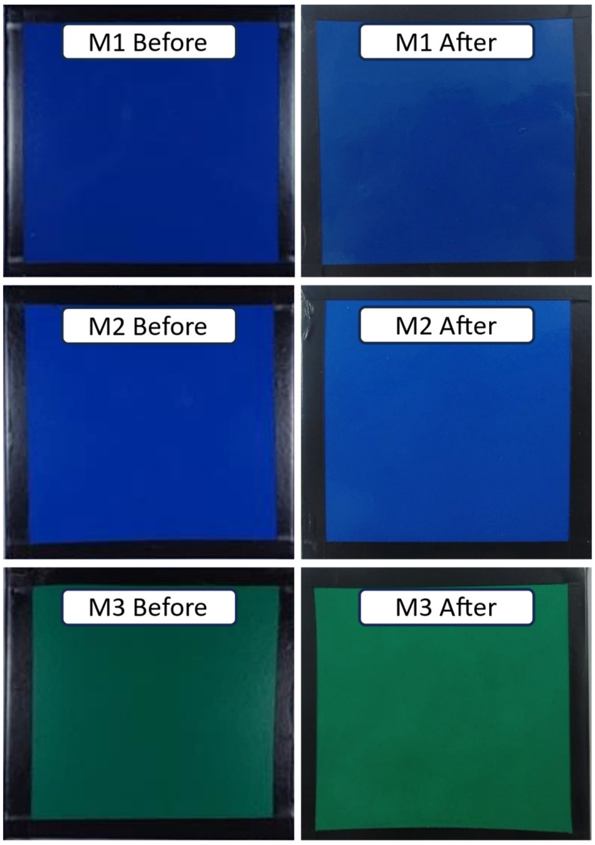


**Figure S4.** Samples of coating systems before and after exposure for 45 months in Antarctica.
